# Design and recruitment of a large-scale cohort study on prevalence, risk factors and impact evaluation of post-COVID-19 condition and its wider long-term social, mental, and physical health impact: The PRIME post-COVID study

**DOI:** 10.3389/fpubh.2022.1032955

**Published:** 2022-12-15

**Authors:** Demi M. E. Pagen, Céline J. A. van Bilsen, Stephanie Brinkhues, Chrissy P. B. Moonen, Maarten Van Herck, Kevin Konings, Casper D. J. den Heijer, Suhreta Mujakovic, Henriëtte L. G. ter Waarbeek, Noortje Bouwmeester-Vincken, Anouk W. Vaes, Martijn A. Spruit, Christian J. P. A. Hoebe, Nicole H. T. M. Dukers-Muijrers

**Affiliations:** ^1^Department of Sexual Health, Infectious Diseases, and Environmental Health, South Limburg Public Health Service, Heerlen, Netherlands; ^2^Care and Public Health Research Institute (CAPHRI), Department of Social Medicine, Maastricht University, Maastricht, Netherlands; ^3^Department of Knowledge and Innovation, South Limburg Public Health Service, Heerlen, Netherlands; ^4^Department of Research and Education, Ciro, Horn, Netherlands; ^5^School of Nutrition and Translational Research in Metabolism (NUTRIM), Department of Respiratory Medicine, Maastricht University Medical Centre+, Maastricht, Netherlands; ^6^The Rehabilitation Research Center (REVAL), BIOMED–Biomedical Research Institute, Faculty of Rehabilitation Sciences, Hasselt University, Diepenbeek, Belgium; ^7^Department of Process and Information Management, Communication and Automation, South Limburg Public Health Service, Heerlen, Netherlands; ^8^Department of Infectious Diseases, North Limburg Public Health Service, Venlo, Netherlands; ^9^Care and Public Health Research Institute (CAPHRI), Department of Medical Microbiology, Maastricht University Medical Centre+, Maastricht, Netherlands; ^10^Care and Public Health Research Institute (CAPHRI), Department of Health Promotion, Maastricht University, Maastricht, Netherlands

**Keywords:** COVID-19, long COVID, post-COVID-19 condition, risk factors, physical health, mental health, socioeconomic impact

## Abstract

**Background:**

Persistent symptoms, described as long COVID or post-COVID-19 condition, pose a potential public health problem. Here, the design and recruitment of the PRIME post-COVID study is described. PRIME post-COVID is a large-scale population-based observational study that aims to improve understanding of the occurrence, risk factors, social, physical, mental, emotional, and socioeconomic impact of post-COVID-19 condition.

**Methods:**

An observational open cohort study was set up, with retrospective and prospective assessments on various health-conditions and health-factors (medical, demographic, social, and behavioral) based on a public health COVID-19 test and by self-report (using online questionnaires in Dutch language). Invited for participation were, as recorded in a public health registry, adults (18 years and older) who were tested for COVID-19 and had a valid Polymerase Chain Reaction (PCR) positive or negative test result, and email address. In November 2021, 61,655 individuals were invited by email to participate, these included all eligible adults who tested PCR positive between 1 June 2020 and 1 November 2021, and a sample of adults who tested negative (2:1), comparable in distribution of age, sex, municipality of residence and year-quarter of testing. New recruitment periods are planned as well. Participants are followed over time by regular follow-up measurements. Data are analyzed using the appropriate data-analyses methods.

**Discussion:**

The PRIME post-COVID study will provide insights into various health-related aspects of post-COVID-19 condition in the context of various stages of the COVID-19 pandemic. Results will inform practical guidance for society, clinical and public health practice for the prevention and care for long-term impact of COVID-19.

**Trial registration ClinicalTrials.gov identifier:**

NCT05128695.

## 1. Introduction

Since 2020, the severe acute respiratory syndrome coronavirus-2 (SARS-CoV-2), causing coronavirus disease 2019 (COVID-19), has been disruptive to societies, globally. By August 2022, over 596 million confirmed cases were counted, accompanied by ~6 million deaths worldwide (1). An acute infection can be diagnosed using Polymerase Chain Reaction (PCR) testing and often presents with symptoms such as fever, cough, tiredness, and loss of taste or smell (2). The majority of the infections progress with mild symptoms and only few (up to a maximum of 20% in elderly) infected individuals require hospitalization (3, 4). Overall, men, people with more comorbidities, older age, and higher body mass index more frequently suffer a severe course of infection, resulting in a higher risk of hospitalization and dying as a consequence of COVID-19 (5, 6).

After great efforts to cope with new waves of SARS-CoV-2 infections globally, the Omicron wave had initially pointed to some hope of ending the pandemic (7), although situations may change quickly. Of an emerging concern are persistent symptoms after a SARS-CoV-2 infection (8–10). These persistent symptoms after COVID-19 are referred to as the patient-initiated term “long COVID” (11), “*a condition whereby affected individuals do not recover for several weeks or months following the onset of symptoms suggestive of COVID-19”* (12). As a global and standardized clinical case definition is lacking and various terminology is used to describe the same condition (13), the World Health Organization (WHO) released the term “post-COVID-19 condition” and a corresponding case definition. The WHO defined “post-COVID-19 condition” in October 2021 as “*a condition that occurs in individuals with a history of probable or confirmed SARS-CoV-2 infection, usually 3 months from the onset of COVID-19 with symptoms, that last for at least 2 months, and cannot be explained by an alternative diagnosis*” (14). Due to the high numbers of SARS-CoV-2 infections worldwide, post-COVID-19 condition poses a potential public health problem with a substantial impact on the socio-economic, emotional, and physical functioning of many individuals.

Currently, the impact of health problems after a SARS-CoV-2 infection are not fully understood, also the prevalence and incidence of post-COVID-19 condition are still unclear. Reasons for this unclarity include difficulties encountered in studying post-COVID-19 condition. In previous scientific studies, this translated into the inclusion of participants without a formal diagnosis of an acute infection, due to the lack of PCR test capacity in the first wave (15), and long-term symptoms after an acute infection (13), due to the lack of a globally accepted case definition at that time before the release of the recent WHO definition. Furthermore, study participants were followed at different time points since infection, participants represented a selected population instead of a population-based population, being included from hospital-based settings (16), medical records (17), peer support groups (9), or social media platforms (18). Furthermore, in many studies, a control or reference group of negatives was lacking (19). Including specific study populations may lead to selection bias and alongside with the lack of harmonization of measures, these limitations hamper the comparison between studies, for instance regarding prevalence, incidence, and impact on health or socioeconomics (20). Furthermore, established risk factors for post-COVID-19 condition are, for example, female sex and pre-existing co-morbidities (21–23), but there are still knowledge gaps on risk factor coherence and possible protecting factors (for example vaccination) for post-COVID-19 condition.

The current study, called Prevalence, Risk factors and Impact Evaluation of post-COVID-19 condition (PRIME post-COVID) study, was set up to gain more insight into the occurrence and impact of post-COVID-19 condition in the community, by including a large-scale population-based PCR positive sample, a negative control group, and follow-up measures for prospective assessment. The findings of this study will contribute to the development of holistic social and health care pathways and support systems. Here, the design and recruitment are described.

## 2. Methods and analysis

The aim of our study is to understand the prevalence, incidence, risk factors and impact of post-COVID-19 condition.

The following study questions will be addressed:

1. What is the prevalence and incidence of post-COVID-19 condition in PCR confirmed SARS-CoV-2 positive adults, by various definitions, accounting for occurrence of symptoms in negative tested adults?
2. What are sociodemographic and medical risk factors (for example vaccination status and virus variant) for having, preserving, and recovering from post-COVID-19 condition?
3. What is the impact of having post-COVID-19 condition on negative health outcomes and socioeconomic aspects, including social, labor, and educational participation?
4. What is the role of having post-COVID-19 condition in maintaining positive health, including social, mental, emotional, and physical health?
5. How can health be promoted in relation to post-COVID-19 condition, using tools from medical and other types of care, in recovery and coping with post-COVID-19 condition?

### 2.1 Design and setting

**Figure 1:**
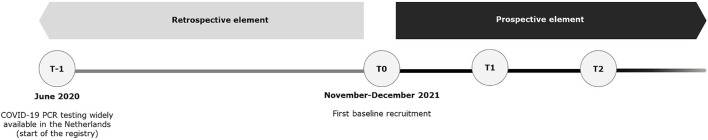
Overview of the prospective and retrospective elements of the PRIME post-COVID study design.

An observational cohort study in positive and negative COVID-19 PCR-tested adults, including a retrospective and prospective element, was conducted to reach the study aims (Figure 1). The design is an open cohort study, meaning that new participants will be included over time. The first inclusion was in November/December 2021.

The study region has ~500,000 adult inhabitants (24) in a mixed urban area. Until now, the South Limburg region presents a comparable number of COVID-19 cases per 100,000 inhabitants compared to the Dutch average (46,115 vs. 45,735, respectively), but the highest national mortality rate of 209 deaths per 100,000 inhabitants, compared to 128 deaths per 100,000 inhabitants nationally (not age-standardized) (25).

The Public Health Service (GGD) South Limburg is the main responsible organizer for regional COVID-19 testing in the study region. Testing was free of charge for all inhabitants in the region from 1 June 2020 onward. Tested persons are standardly registered in the Dutch COVID-19 database CoronIT. The study population was approached to participate from this registry.

The Strengthening the Reporting of Observational studies (STROBE) checklist was used in reporting this study (Supplementary Table 1).

### 2.2 Study population

The target population consisted of adults tested for COVID-19 at the GGD South Limburg with a valid PCR test result (positive/negative). We here describe the recruitment of people tested between 1 June 2020 and 1 November 2021. The GGD South Limburg tested 472,982 adults in this period. Eligible for participation were tested adults (18+ years) with a registered email address available. Of eligible adults, all people with a positive COVID-19 PCR test, and a sample of people with a negative COVID-19 PCR test were selected. All positives who met the inclusion criteria were invited to obtain a representative sample of all positives in the test registry. A reasonable sample of ~20,000 negatives were additionally included (ratio 2:1). Regarding the positives, when a person had tested positive multiple times, the record of the first positive COVID-19 test was used in the analyses, as we intended to include data on the first exposure. Regarding the negatives, a sample of people with a negative PCR test were randomly selected in each strata with a distribution that matched (1:2) positives; strata included combinations of age (18–20, 20–30, 30–40, 40–50, 50–60, 60–70, 70–80, and 80+ years), sex (man, woman), year-quarter of 2020 and 2021 in which PCR test was performed, and municipality of residence (*n* = 16) (Table 1). When tested negative multiple times, the information regarding the last negative COVID-19 test was used to have the greatest assurance that an invited control was not infected up to the point of inclusion. This procedure allowed to include a group of negatives, similar to the positives regarding sociodemographic and timing of testing.

**Table 1:** Descriptives of invitees of the prevalence, risk factors and impact evaluation of post-COVID-19 condition (PRIME post-COVID) study.

**Test result, *n* (%)**	**Invitees** ***n*** = **61,655**	
	**Negative**	**Positive**
	19,875 (32.2)	41,780 (67.8)
**Sex**, ***n*****(%)**
Men	9,917 (49.9)	18,794 (45.0)
Women	9,958 (50.1)	22,986 (55.0)
**Age**, ***n*****(%)**
18–20 years	1,211 (6.1)	3,519 (8.4)
20–30 years	3,719 (18.7)	9,367 (22.4)
30–40 years	3,202 (16.1)	6,823 (16.3)
40–50 years	2,991 (15.1)	6,560 (15.7)
50–60 years	3,678 (18.5)	7,816 (18.7)
60–70 years	3,098 (15.6)	4,697 (11.2)
70–80 years	1,525 (7.7)	2,252 (5.4)
80+ years	451 (2.3)	746 (1.8)
**Year-quarter of test**, ***n*****(%)**
2nd 2020^*^	361 (1.8)	49 (0.1)
3rd 2020	2,267 (11.4)	716 (1.7)
4th 2020	5,718 (28.8)	12,060 (28.9)
1st 2021	4,255 (21.4)	9,341 (22.4)
2nd 2021	3,635 (18.3)	9,561 (22.9)
3rd 2021	2,483 (12.5)	6,106 (14.6)
4th 2021^**^	1,156 (5.8)	3,947 (9.4)

^*^From June 2020.

^**^Until November 2021.

### 2.3 Recruitment

Emails to invitees were send between 17 and 25 November 2021. Invited for participation were 41,780 PCR COVID-19 positives and 19,875 negatives from test records registered in the registry. Invitees received an email containing the invitation for participation, study information with a link to the website (containing the participant information and email address in case of questions), and a weblink to the online questionnaire.

Participants needed to be able to understand, read and write the Dutch language, as all materials were provided in Dutch.

Digital informed consent was asked before the questionnaire started. Further digital informed consent was asked to link data from the questionnaire to the registry data (age, sex, municipality, date of test, and test result).

A reminder was sent 1 and 3 weeks after the invitation to invitees who did not respond to the invitation or started but not completed the questionnaire. The questionnaire was made available using the MWM2 application of market research platform Crowdtech (ISO 27001 certified).

### 2.4 Inclusion

Participation started after informed consent was obtained online. Filling out the baseline questionnaire took ~30–45 min. To motivate completion of the questionnaire, participants could stop interim and complete the questionnaire at a later time.

### 2.5 Data collection

Data are collected by online self-administered questionnaires. The baseline questionnaire could be filled in between 17 November 2021 until 9 January 2022. The questionnaire contained a range of factors, including health-factors and health-conditions (Table 2). At baseline, information on the current situation as well as historical information (such as symptoms between the test and the current date) was collected from the participants. Several in-depth questions were only deemed relevant for positives or negatives, and thus not asked to all participants to minimize unnecessary load.

**Table 2:** Topics and questions implemented in the questionnaire of the baseline recruitment of the prevalence, risk factors and impact evaluation of post-COVID-19 condition (PRIME post-COVID) study.

**Topic**	**Questions**	**Asked to**
Demographics	Sex	All
	Year of birth	All
	Postal code	All
	Residential situation	All
	Relationship status	All
	Level of education	All
	Country of birth	All
COVID-19 test data	Test result	All
	Test date	All
	Vaccination status	All
	Vaccination date(s)	All
	Vaccination type(s)	All
	Presence of antibodies	Negatives
Experienced symptoms	Symptoms when tested	All
	Symptoms now	All
	Severity of symptoms	All
	Duration of symptoms	All
	Course of symptoms	Positives
	Subjective assessment of degree of recovery	Positives
	Subjective assessment of having post-COVID-19 condition	Positives
Use of professional medical care	Treatment when tested (hospitalization, duration, and oxygen use)	Positives
	Received care since test	All
	Reason for received care	Positives
Lifestyle	Smoking and substance	All
	Physical activity [international physical activity questionnaire; IPAQ (26)]	All
Experienced physical and mental health	Underlying disease	All
	Origination of underlying disease	All
	Overall general health	All
	Quality of life [EQ-5D-5L (27)]	All
	Dyspnoea [modified Medical Research Council; mMRC (28)]	All
	Fatigue [checklist individual strength—subscale subjective fatigue; CIS (29)]	All
	Mental fatigue [self-constructed based on CIS-Fatigue (30)]	All
	Coping [cognitive emotion regulation questionnaire; CERQ (31)]	All
	Trauma [PTSD checklist for the DSM-5; PCL-5 (32)]	Positives
	Depression [patient health questionnaire-9; PHQ-9 (33)]	All
	Loneliness [De Jong-Gierveld (34)]	All
Labor participation	Labor participation [work productivity and activity impairment; WPAI (35)]	All
Social participation	Membership to clubs	All
Structural and functional social network characteristics assessed with name generator method (36)	Type and number of network members (family, friends, acquaintances, neighbors, colleagues, clubmates, caregivers, others)	All
	Social support of each member	All
	Type of relation with each member	All
	Social strain	All
	Age of each member	All
	Sex of each member	All
	Residential distance of each member	All
	Type of contact with each member	All

Question asked to participants tested positive or negatives for COVID-19 only was done using referral based on self-reported test result in questionnaire.

Linkage of questionnaire and registry was done by unique email address, by the data manager. For data storage and analyses, each participant has a unique study code under which all data (questionnaires and registration data) are handled.

#### 2.5.1 Prospective element: Follow-up

Follow-up measures are planned roughly each 6 months, with the option of in-between measures to assess emerging topics. All questionnaires include topics on demographics, COVID-19 test data, experienced symptoms, use of care, lifestyle, experienced physical and mental health, and labor and social network and social participation (Table 2). Additions will be made to the follow-up questionnaires, based on new insights in literature and emerging findings of data analyses.

### 2.6 Data analysis

#### 2.6.1 Primary outcomes in analysis

The time frame for collecting the primary outcomes (summarized below) runs from the baseline questionnaire up to at least 2 years thereafter, assessed at regular intervals.

The main primary outcome:

- Post-COVID-19 condition: the symptoms that are defined by the WHO-definition of post-COVID-19 condition, as well as other types of definition. These include a range of symptoms measured at various time points, for example, the presence of at least one symptom 3 months after testing or when filling in the questionnaire, and experiencing of dyspnea (modified Medical Research Council; mMRC) or fatigue (Checklist Individual Strength—subscale subjective fatigue; CIS).

Other primary outcomes include:

- Mental health: negative health outcomes related to mental health such as depression (assessed by PHQ-9 scale) and trauma.
- Social health: negative health outcomes related to social health, such as loneliness (assessed by De Jong-Gierveld scale).
- Physical health: negative health outcomes related to physical health such as non-communicable chronic diseases (assessed upon self-report on a checklist of conditions present), health index/quality of life (assessed by EQ-5D-5L) and physical activity (assessed by IPAQ).
- Positive health: experienced general health (assessed by 5-point scale ECHI).

#### 2.6.2 Certainty assessment

**Figure 2:**
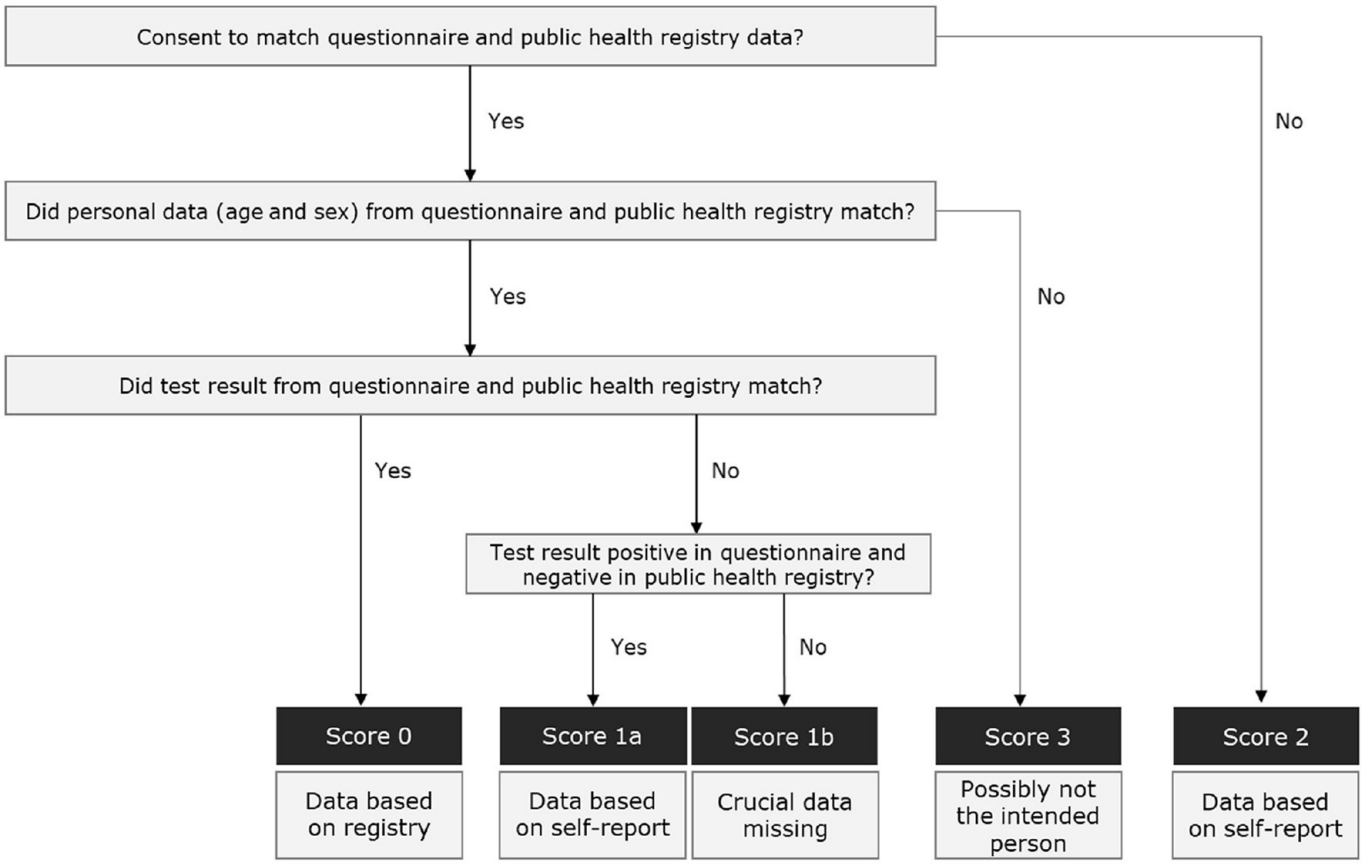
Decision tree to ascertain certainty assessment scores based on self-reported and public health registry data.

Data on age, sex, and test result provided in the questionnaire was linked back to this data as recorded in the registry from the corresponding email address, for purposes of certainty assignment, i.e., the likelihood that the respondent indeed was the intended invitee (Figure 2). Respondents were assigned a certainty score 0 (most likely the intended invitee) when respondents consented to link questionnaire and registry data on age, sex, and test result, and this data was the same. Score 1 was given when only the test result was discrepant. Participants who did not consent to link questionnaire and registry data were assigned with score 2. Score 3 was assigned to respondents who did not match on sex or age, representing probably another person than the intended invitee.

Furthermore, it cannot be formally ruled out that the negative PCR-tested participants, who also did not report COVID-19 antibodies pre-vaccination, might include some COVID-19 positive cases. The absence of SARS-CoV-2 antibodies does not guarantee that an infection did not take place since some people (often mild cases) do not show sero-conversion and also sero-reconversion occurs over time (37–40). We will take reported antibody status in PCR negatives into account when estimating the prevalence of experienced symptoms.

#### 2.6.3 Missing data and data cleaning

The questionnaire started with collection of clinical and demographical data. Participants who did not fill in these topics had insufficient data for any of the planned analyses. The remaining participants were categorized based on extent to which they completed the rest of the questionnaire. All questions were mandatory, therefore no missing data had to be handled and no imputation methods were used.

### 2.7 Statistical analysis

The obtained data will be analyzed using descriptive statistics, such as on prevalence of post-COVID-19 condition including 95% confidence intervals. To assess prevalence, participants tested negative are also presented, providing some insight of “background risk,” as symptoms of post-COVID-19 condition may be nonspecific and present in the general population (41, 42). Prevalence of reported symptoms in post-COVID-19 condition participants will be compared to the prevalence in PCR negatives. Post-COVID-19 incidence will be estimated when follow-up data is available, by determining the proportion of newly diagnosed participants (since baseline) meeting the post-COVID-19 condition criteria. Multivariable models are used to test for the likelihood of (long-term) symptoms in positive vs. negative participants, adjusted for possible confounders. Possible confounders include age, sex, underling disease, severity of COVID-19, and year-quarter when tested. Risk factor analyses, with post-COVID-19 condition as outcome, will be performed using multivariable logistic regression models. Various sensitivity analyses will be performed, e.g., by only including participants with highest certainty score 0. These are examples of analyses to answer a main study question. Appropriate statistical analyses will be performed to answer all study questions.

## 3. Discussion

The current paper outlined the design and recruitment of an observational population-based open cohort study in positive and negative COVID-19 PCR-tested adults. The cohort includes a retrospective and prospective element, to understand the prevalence, risk factors, and impact of post-COVID-19 condition.

The findings of this study will contribute to the further characterization of post-COVID-19 condition and provide new insights into the occurrence, recovery, and physical, emotional, social, and economic impact of post-COVID-19 condition. Furthermore, it will help increase awareness and recognition of post-COVID-19 condition and contribute to the development of holistic social and health care pathways and support systems.

### 3.1 Strengths and limitations

A major strength of this cohort study is the design comprising of a retrospective and prospective element. In general, including a large population-based sample is a strength, as large sample sizes promote precision and reliability of prevalence estimates and associations between post-COVID-19 condition and risk factors (43). By including a population-based sample we were able to minimize selection bias toward the inclusion of specific groups, such as hospital-based populations. Moreover, including PCR test-negative individuals to determine long-term symptoms and impact of the COVID-19 pandemic in general, made it possible to identify symptoms and impact accountable to a SARS-CoV-2 infection. Matching test-negatives to test-positives on key characteristics (i.e., sex, age, and year-quarter of testing) when sampling limits possible confounding by accounting for the distribution of these characteristics. Finally, a wide range of physical, emotional, social, and behavioral aspects will be mapped using extensive questionnaires, including multiple standardized and validated questions on diverse topics. Doing so enables us to approach post-COVID-19 condition holistically, and to correct future analysis for several confounding factors. By the comprehensive assessment in the current study, post-COVID-19 condition will be classified by several definitions put forward in the literature. These definitions will be compared with each other, and with the established case definition provided by the WHO as well.

The main limitation resides in the use of self-reported online questionnaire data. No additional clinical assessment or investigations on symptoms and physical fitness could be performed. This method of data collection (i.e., using self-administered questionnaires) is always susceptible to information bias. As expected with large-scale population-based studies, a number of the invitees might not respond. Invitees being less digitally skilled are hindered to participate without assistance, which might result in selection (non-response) bias and reduced external validity. The same holds for invitees who were unable to read or understand the Dutch language, as all materials were provided in Dutch. Differential selection bias might occur when the chance of participation is higher in test-positives vs. test-negatives, when positives believe to suffer from long-term symptoms since their infection. Possible over- and under representations of specific subgroups in the eventual study population will be considered when estimating post-COVID-19 condition prevalence, for example by weighing these subgroups according to the distribution in the invited population to enhance representativeness and thereby external validity. Overrepresentations may affect internal validity when non-participation of specified subgroups is related to both exposure and outcome. We will therefore study whether the association between risk factors and post-COVID-19 condition differs (i.e., heterogeneity of the effect) in different subgroups. Lastly, we acknowledge the possible limited generalizability of our results, due to the focus on the southern region of the Netherlands.

Despite these general strengths and limitations of the design, the retrospective and prospective elements both have their own strengths and limitations to be discussed.

#### 3.1.1 Retrospective element

The retrospective aspect of the study design allows us to obtain crucial data on health status and required professional care when tested and the months thereafter, until filling out the questionnaire.

In this respect, the main limitation to be mentioned is the possibility of recall bias, which mainly occurs when participants were tested a long time ago. Recall bias might be differential, for example when positives recall experienced symptoms different compared to negatives. Nevertheless, the risk of misclassification bias in the test result (i.e., the exposure data) and possible confounders (i.e., sex, age, and year-quarter of testing) was diminished by linking the data from the questionnaire and public health registry data for the majority of the participants.

#### 3.1.2 Prospective element

The major strength of the prospective assessments is the opportunity to study changes in outcomes and determinants before, during, and after (re)infection. Causal hypotheses can be considered, as the order of observations is equal to the natural course of events over time. Besides, overall PCR test data (from June 2020 onwards) can be accessed from the public health registry as well, to gain a reliable estimation of total exposure (i.e., the total number of positive tests/re-infections). Furthermore, the inclusion of new participants tested in later calendar periods (open cohort design) is of great value to study the prevalence of post-COVID-19 condition in the context of new emerging virus variants. In addition, it is predicted that the proportion of test-negative controls declines over time, due to acquired infection. The prospective aspect enables us to include supplemental controls to maintain a sufficient number of test-controls.

Attrition bias is the major limitation of the prospective element to be acknowledged. Selective withdrawal might occur when participants are physically unable to complete follow-up questionnaires, due to severe illness for example. This disproportional attrition threats the internal validity and might lead to underestimation or overestimation of prevalence estimates or associations (43). In general, we will consider the described limitations in the interpretation of our results, and when possible, take them into account in analysis.

To conclude, the PRIME post-COVID longitudinal study design holds several considerable strengths, such as the mapping of a wide variety of physical, emotional, social, and behavioral aspects, and including a population-based sample additionally comprising PCR negatives. Results of our study will contribute to the further characterization of post-COVID-19 condition and thereby inform practical guidance for society, and clinical and public health practice for the prevention and care for long-term impact of COVID-19.

## 4. Ethics and dissemination

### 4.1 Ethical considerations

The Medical Ethical Committee of Maastricht University Medical Center+, Maastricht Netherlands waived this study (METC2021-2884), as the Medical Research Involving Human Subjects Act (WMO) did not apply to this study. The study is additionally registered at ClinicalTrials.gov Protocol Registration and Results System (NCT05128695).

Invitee contact details were retrieved from regular infectious disease control test activities from our medical records. Invitees received an email containing the invitation for participation and a link to the online questionnaire. Information about the study was provided, including the link to the study website, containing the participant information. Voluntary digital informed consent was asked before the questionnaire started.

After participation, data were fully de-identified for further analysis. The study protocol was exempt from formal medical-ethical approval under prevailing laws in the Netherlands, as it concerns an observational study using anonymous questionnaire data only (as stated by the National Central Committee for Human Studies: www.ccmo.nl and in the conduct of good behavior in research). As such, no additional administrative permissions were compulsory to use the required data, as it was owned by our own public health service. Additionally, a Data Protection Impact Assessment (DPIA) was conducted as determined in the General Data Protection Regulation (GDPR), to take appropriate precautionary measures to limit privacy risks of data processing.

### 4.2 Dissemination

Key results of the study will be communicated to the participants using a comprehensible report written for a lay audience. The findings of our study will be published in peer-reviewed journals and presented in scientific meetings and conferences as well.

## Ethics statement

Ethical review and approval was not required for the study on human participants in accordance with the local legislation and institutional requirements. The patients/participants provided their written informed consent to participate in this study.

## Author contributions

DP, CB, SB, CM, MV, KK, CHe, AV, MS, CHo, and ND-M designed the study and developed the questionnaire. DP wrote the first draft of the manuscript. All authors revised the manuscript critically for important intellectual content, approved the definitive version, and agreed to be accountable for all aspects of the manuscript.
